# P-2286. Invasive Fungal Infections in Patients Treated with Bruton Tyrosine Kinase Inhibitors: a Systematic Review and Meta-Analysis

**DOI:** 10.1093/ofid/ofae631.2439

**Published:** 2025-01-29

**Authors:** Tanaporn Meejun, Karan Srisurapanont, Lucy X Li, Shmuel Shoham, Robin K Avery, John W Baddley, Veronica Dioverti, Olivia S Kates, Nitipong Permpalung

**Affiliations:** Faculty of Medicine, Chulalongkorn University, Chom thong, Krung Thep, Thailand; Faculty of Medicine Chiang Mai university, Chiang Mai, Chiang Mai, Thailand; Johns Hopkins University, Baltimore, Maryland; Johns Hopkins University School of Medicine, Baltimore, Maryland; Johns Hopkins, Baltimore, Maryland; Johns Hopkins University School of Medicine, Baltimore, Maryland; Johns Hopkins, Baltimore, Maryland; Johns Hopkins University, Baltimore, Maryland; Johns Hopkins University School of Medicine, Baltimore, Maryland

## Abstract

**Background:**

Controversy surrounds the true incidence of invasive fungal infections (IFIs) in patients on Bruton tyrosine kinase inhibitors (BTKIs) due to incomplete epidemiological data. This systematic review and meta-analysis evaluated the prevalence and risk factors of IFIs in BTKI-treated patients.

**Figure 1.**
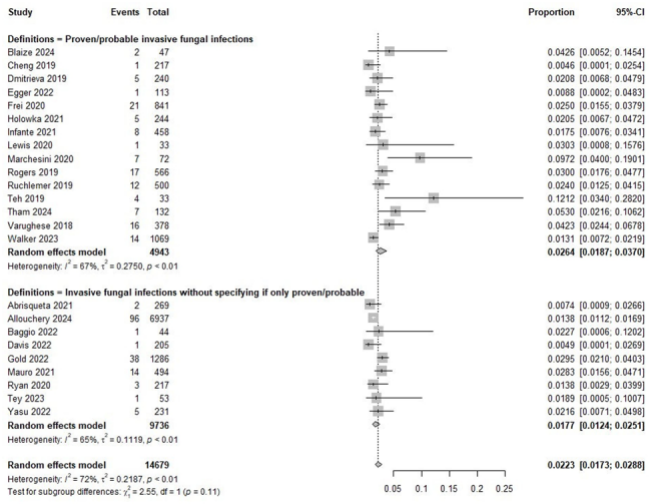
Forest plot for the prevalence of invasive fungal infections in patients treated with Bruton tyrosine kinase inhibitors

This study was registered under PROSPERO (CRD42024523198). The prevalence was calculated using a generalized linear mixed model random-effects meta-analysis and presented as effect sizes with 95% confidence intervals (CIs). IFIs were defined according to The European Organization for Research and Treatment of Cancer and the Mycoses Study Group Education and Research Consortium (EORTC/MSGERC) consensus definitions.

**Methods:**

Studies up to April 17, 2024, from Pubmed, Scopus, and Embase, including observational studies reporting prevalence and/or associated risk factors of IFIs with at least 30 BTKI-treated patients, were searched and analyzed. IFIs were defined according to standard definitions. The prevalence of IFIs and odds ratio (ORs) for the risk factors associated with IFIs were reported alongside 95% confidence intervals (CIs).

**Figure 2.**
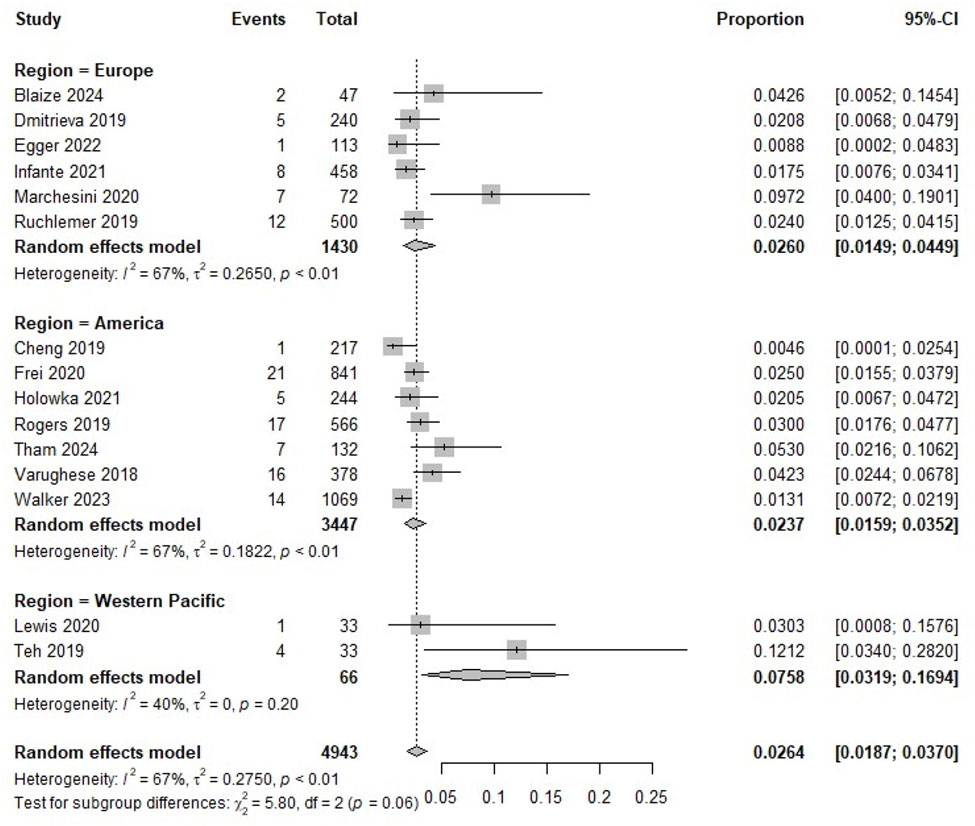
Forest plot for the prevalence of proven/probable invasive fungal infections in patients treated with Bruton tyrosine kinase inhibitors according to the World Health Organization regions

**Results:**

This study included 14,679 patients across 24 studies (13,981 with hematologic malignancies and 67 with chronic graft-versus-host disease). Of these, 15 studies specifically included only proven/probable IFIs (ppIFIs) (Figure 1 and Table 1). The overall IFI prevalence was 2.23% (95% CI 1.73-2.88%), and for ppIFIs, was 2.64% (95% CI 1.87-3.70%). The median durations from BTKI initiation to ppIFI onset ranged from 24-186 days. The highest prevalence of ppIFIs was in patients with mantle cell lymphoma (7.60%, 95% CI 3.14-17.26%), followed by chronic lymphocytic leukemia (3.01%, 95% CI 1.93-4.66%), and Waldenström's macroglobulinemia (2.18%, 95% CI 0.33-12.96%). Regional ppIFI prevalence was the highest in Western Pacific (7.58%, 95% CI 3.19-16.94%), followed by Europe (2.60%, 95% CI 1.49-4.49%) and America (2.37%, 95% CI 1.59-3.52%) (Figure 2). The most common ppIFIs were invasive aspergillosis (1.78%, 95% CI 1.24-2.57%), followed by pneumocystosis (0.80%, 95% CI 0.16-3.81%), invasive candidiasis (0.40%, 95% CI 0.14-1.16%), and cryptococcosis (0.33%, 95% CI 0.19-0.56%). Corticosteroid use was significantly associated with ppIFIs (OR 5.03, 95% CI 2.31-10.92) (Figure 3).

**Figure 3.**
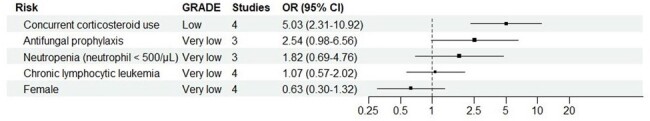
Forest plot for risk factors associated with proven/probable invasive fungal infections in patients treated with Bruton tyrosine kinase inhibitors GRADE = Grading of Recommendations Assessment, Development and Evaluation certainty of evidence; OR = odds ratio. Odds ratios were directly extracted from regression analysis or manually calculated by comparing associated factors between patients with and without invasive fungal infections.

**Conclusion:**

In conclusion, the prevalence of IFIs in patients treated with BTKIs (mostly ibrutinib) reaches 2%, and up to 7.6% in those with mantle cell lymphoma, indicating a significant risk. Antifungal prophylaxis should be customized based on individual underlying diseases and steroid use.

**Table 1.**
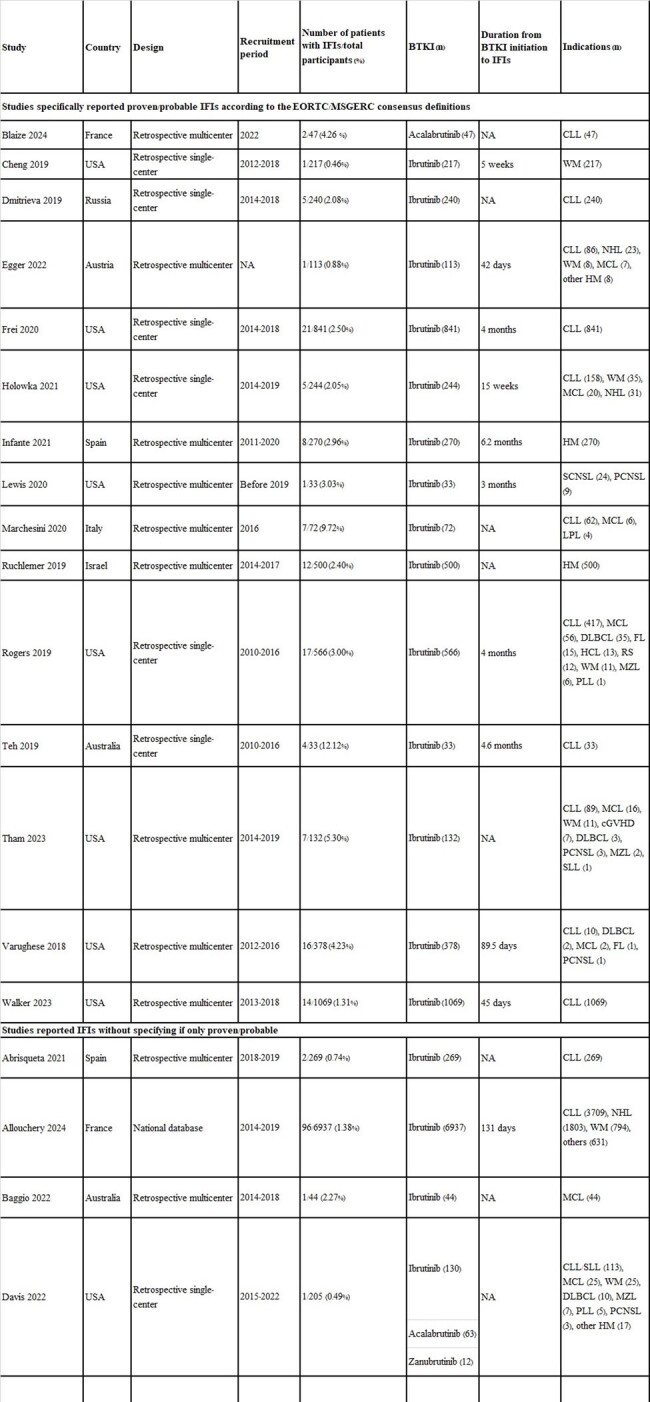
Characteristics of included studies

BTKI = Bruton tyrosine kinase inhibitor; cGVHD = chronic graft-versus-host disease; CLL = chronic lymphocytic leukemia; DLBCL = diffuse large B-cell lymphoma; EORTC/MSGERC = the European Organization for Research and Treatment of Cancer and the Mycoses Study Group Education and Research Consortium; FL = follicular lymphoma; HCL = hairy cell leukemia; HM = hematologic malignancies; LPL = lymphoplasmacytic lymphoma; MCL = mantle cell lymphoma; MZL = marginal zone lymphoma; NA = not available; NHL = non-Hodgkin’s lymphoma; PCNSL = primary central nervous system lymphoma; PLL = prolymphocytic leukemia; RS = Richter’s syndrome; SCNSL = secondary central nervous system lymphoma; SLL = small lymphocytic lymphoma; USA = United States of America; WM = Waldenström's macroglobulinemia

**Disclosures:**

Shmuel Shoham, MD, F2G: Grant/Research Support Veronica Dioverti, MD, AlloVir: Grant/Research Support|Regeneron: Advisor/Consultant|Regeneron: Grant/Research Support Nitipong Permpalung, MD, MPH, CareDx: Grant/Research Support|Cidara Therapeutics: Grant/Research Support|ClearView: Advisor/Consultant|IMMY Diagnostics: Grant/Research Support|Merck: Grant/Research Support|Pearl Diagnostics: Grant/Research Support|Pulmicide: Advisor/Consultant|Scynexis: Grant/Research Support

